# Proteoglycan Combined with Hyaluronic Acid and Hydrolyzed Collagen Restores the Skin Barrier in Mild Atopic Dermatitis and Dry, Eczema-Prone Skin: A Pilot Study

**DOI:** 10.3390/ijms221910189

**Published:** 2021-09-22

**Authors:** Young In Lee, Sang Gyu Lee, Jemin Kim, Sooyeon Choi, Inhee Jung, Ju Hee Lee

**Affiliations:** 1 Department of Dermatology & Cutaneous Biology Research Institute, Yonsei University College of Medicine, Seoul 03722, Korea; ylee1124@yuhs.ac (Y.I.L.); dltkdrb5658@yuhs.ac (S.G.L.); JEMIN89ZZ@yuhs.ac (J.K.); CHOISY429@yuhs.ac (S.C.); 2 Scar Laser and Plastic Surgery Center, Yonsei Cancer Hospital, Seoul 03722, Korea; 3 Global Medical Research Center, Seoul 06526, Korea; ihjung@gmrc.co.kr

**Keywords:** dry skin, eczema, proteoglycan, skin barrier, liposome, hydration

## Abstract

Dry and eczema-prone skin conditions such as atopic dermatitis and xerotic eczema primarily indicate an impaired skin barrier function, which leads to chronic pruritus. Here, we investigated the effects of a novel emollient containing H.ECM^TM^ liposome, which contains a soluble proteoglycan in combination with hydrolyzed collagen and hyaluronic acid. A prospective, single-arm study was conducted on 25 participants with mild atopic dermatitis or dry skin to assess the hydration and anti-inflammatory effect of the novel emollient applied daily over four weeks. All efficacy parameters, including itching severity, transepidermal water loss, and skin hydration, improved significantly after four weeks. The in vitro and ex vivo studies confirmed the restoration of the skin’s barrier function. The study revealed the clinical and laboratory efficacy of H.ECM^TM^ liposome in reducing itching and improving the skin’s barrier integrity. Thus, the use of H.ECM^TM^ liposome can be considered a therapeutic option for dry and eczema-prone skin.

## 1. Introduction

Skin, the largest organ, is in constant contact with the external environment and forms a protective barrier against water loss. Usually, young and well-moisturized normal skin is less susceptible to environmental injuries and infection. Therefore, itching and inflammation associated with dry skin and eczema are less likely to occur [1]. Dry skin is a common condition in which the capacitance of the skin is reduced owing to a decrease in skin barrier function, affecting approximately 20% of children and 30–75% adults over the age of 60 years [2]. Dry and eczema-prone skin is frequently the result of ageing or specific conditions such as atopic dermatitis or hemodialysis [1]. Atopic dermatitis is a representative, chronic, relapsing inflammatory skin disease characterized by itching, eczema, and xerosis. Increasing evidence suggests that skin barrier dysfunction is an etiological contributor to the development of atopic dermatitis [3]. The skin can also lose epidermal barrier function when irradiated by ultraviolet light (UV) light and shows markedly increased transepidermal water loss (TEWL), which eventually results in dry, photoaged skin [4,5].

Proteoglycan (PG) is a constituent of the extracellular matrix and is widely distributed in connective tissues such as skin, bone, cartilage, and the vascular wall forming complexes with collagen, fibronectin, laminin, and hyaluronic acid (HA) [6]. It has been shown that PG extracted from salmon nasal cartilage is a potent suppressor of inflammatory responses induced by heat-killed *Escherichia coli* in mouse macrophages [7]. The PG is usually extracted from salmon nasal cartilage, a byproduct of salmon processed foods, making its production environmentally advantageous. PG modulates the inflammatory state of infected wounds and promotes wound healing in mice [8]. PGs bind with HA to form multi-molecular aggregates in cartilage and provide matrix binding sites and cell surface receptors for growth factors such as fibroblast growth factors [9]. PGs also possess epidermal growth factor-like domain, which are modular components of larger proteins with growth factor activity [9]. Therefore, PGs may enhance the skin barrier function and wound healing by playing a role in anti-inflammatory function, modulating the proliferative activity of fibroblasts, and promoting matrix binding by growth factors.

Various skincare products have been developed to maintain and improve the skin barrier function and increase its water content. In this study, we investigated the efficacy of the novel nanoliposome formulation comprising soluble proteoglycan combined with HA and hydrolyzed collagen in restoring the skin barrier function in patients with mild atopic dermatitis and dry skin. We investigated whether soluble proteoglycan synergized the effects of HA and hydrolyzed collagen, both of which are the well-known major constituents of the extracellular matrix that improve skin water content, TEWL, and skin elasticity [10,11]. We performed a prospective, single-arm clinical trial on 25 Asian patients with mild atopic dermatitis or dry skin to evaluate the effects of the H.ECM^TM^ liposome-containing emollient comprising the soluble proteoglycan mixed with HA and hydrolyzed collagen. Moreover, in vitro and human ex vivo experiments were conducted to explore the pathophysiologic mechanism by which the H.ECM^TM^ liposome-containing emollient enabled the restoration of skin barrier function and hydration.

## 2. Results

### 2.1. Patient Characteristics

The clinical study was conducted on 25 Asian patients, aged 19 years or older, with mild atopic dermatitis and dry skin. The mean age of the participants was 40.7 ± 11.5 years (range, 20–56 years). Four participants were male, while 21 participants were female. Seven participants were diagnosed with mild atopic dermatitis, while the remaining 18 patients had xerotic skin with a xerosis assessment scale (XAS) score greater than 2. Two of the participants dropped out due to non-compliance. In the final analysis, data from 23 participants were collected and analyzed. The patients’ demographic data are presented in Supplementary Table S1.

### 2.2. Clinical Efficacy of a Topical Proteoglycan Product Combined with HA and Collagen

**Figure 1:**
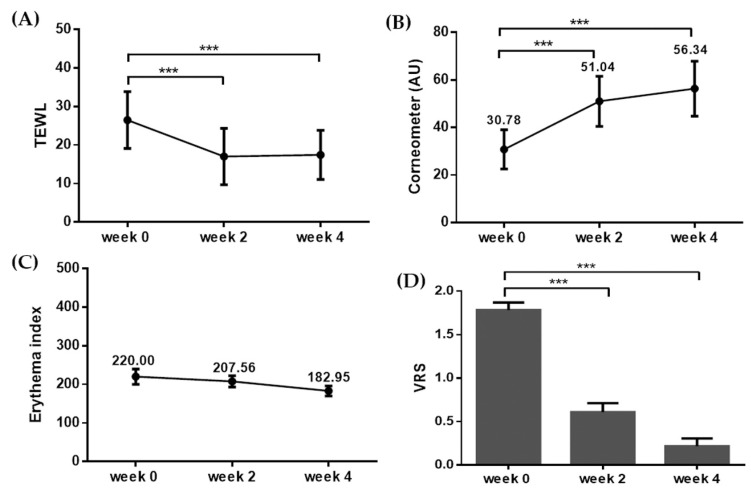
Changes in the biophysical parameters and itching after four-week application of the H.ECM^TM^ liposome-containing emollient. The evaluations for (**A**) transepidermal water loss (TEWL), (**B**) skin hydration (using Corneometer), (**C**) erythema index, and (**D**) VRS (verbal rating scale) showed improvements after the application of the H.ECM^TM^ liposome-containing emollient. *** *p* < 0.005.

The participants applied the H.ECM^TM^ liposome-containing emollient twice daily onto their skin lesions for 4 weeks. The improvement of the skin barrier function was measured primarily by evaluating TEWL. The TEWL decreased significantly from 26.47 ± 7.36 to 17.00 ± 7.30 at week 2, and 17.43 ± 6.35 at week 4 (Figure 1A, *** *p* < 0.005, *t*-test). Moreover, the level of skin hydration measured using the Corneometer showed significant improvement at weeks 2 and 4 over the baseline. The baseline skin hydration was 30.78 ± 8.24, and it increased to 51.04 ± 10.54 and 56.34 ± 11.57 at weeks 2 and 4, respectively (Figure 1B, *** *p* < 0.005, *t*-test). The degrees of erythema were assessed according to the erythema index using Mexameter, and the index values were 220.00 ± 95.55, 207.56 ± 70.18, and 182.95 ± 62.91 at baseline, week 2, and week 4, respectively (Figure 1C). However, the difference in the degree of erythema before and after the treatment was not statistically significant.

**Figure 2:**
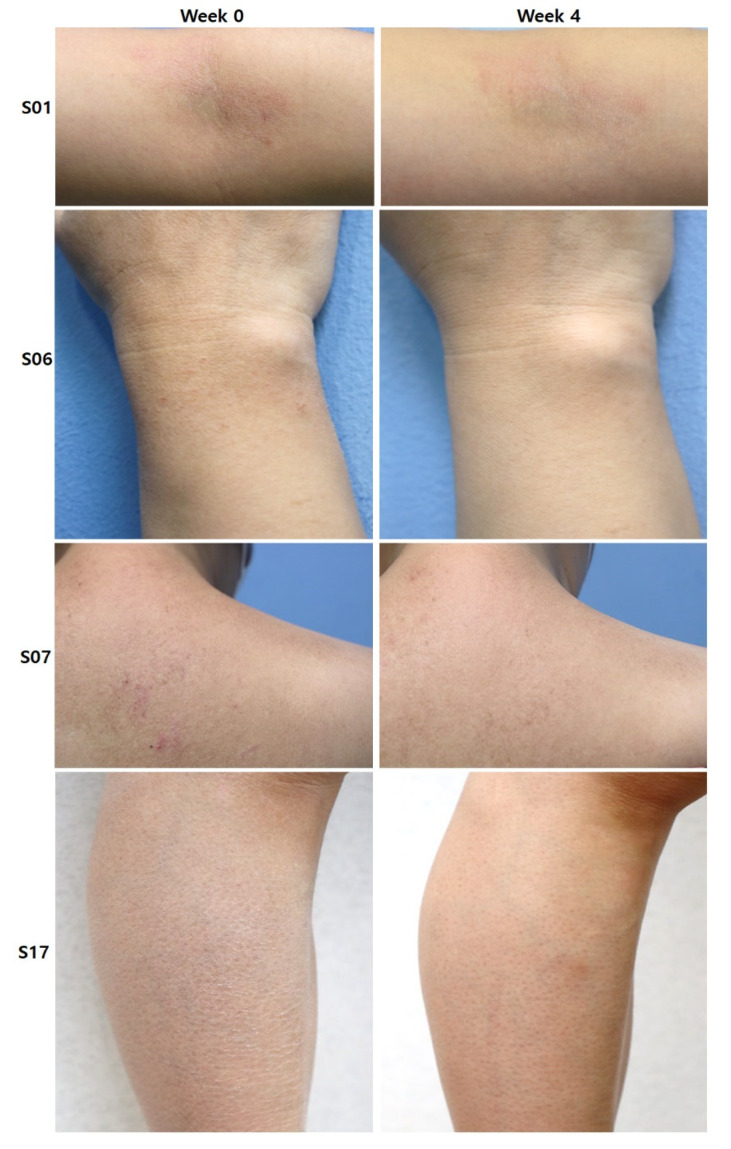
Images showing improvement of skin hydration and erythema after four weeks of H.ECM^TM^ liposome-containing emollient application (subject examples S01, S06, S07, and S17).

The primary outcome for comparing the symptom severity before and after treatment was assessed by the difference in the verbal rating scale (VRS) on itching. Compared to the baseline average VRS of moderate itching (1.78 ± 0.42), VRS decreased significantly to mild itching (0.60 ± 0.50) at week 2, and mild to almost no itching (0.23 ± 0.42) at week 4 (Figure 1D, *** *p* < 0.005, *t*-test). The average global improvement assessment score (GIS) at the same time points as assigned by three blinded, independent investigators was 1.84 ± 0.76 (marked improvement), while the average GIS by the participants was 1.48 ± 0.51 (near-total improvement; Figure 2).

No adverse events were observed during the four-week study period. None of the participants dropped out of the study because of adverse events, suggesting that the topical H.ECM^TM^ liposome formulation was safe to use.

### 2.3. In Vitro Evaluation of H.ECM^TM^ Liposome and Its Efficacy on Skin Barrier Function and Wrinkles

To measure if H.ECM^TM^ liposome-containing emollient was cytotoxic, an MTT assay was performed using a mouse macrophage (RAW 264.7), human epidermal keratinocyte (KC), and human dermal fibroblast (HDF) cells. All cell lines were treated with 0.01%, 0.05%, 0.1%, 0.5%, and 1% H.ECM^TM^ liposomes, and the results of the MTT assay are shown in comparison with those of the control cells. Additionally, the cytotoxicity of lipopolysaccharide (LPS; 0.5–50 μg/mL) was evaluated in HDF cells. The results of the MTT assay for each cell line are shown in Supplementary Figure S1. Based on the results, 0.05% H.ECM^TM^ liposome treatment was used in the real-time quantitative reverse transcription polymerase chain reaction (qRT-PCR) and immunofluorescence staining experiments.

**Figure 3:**
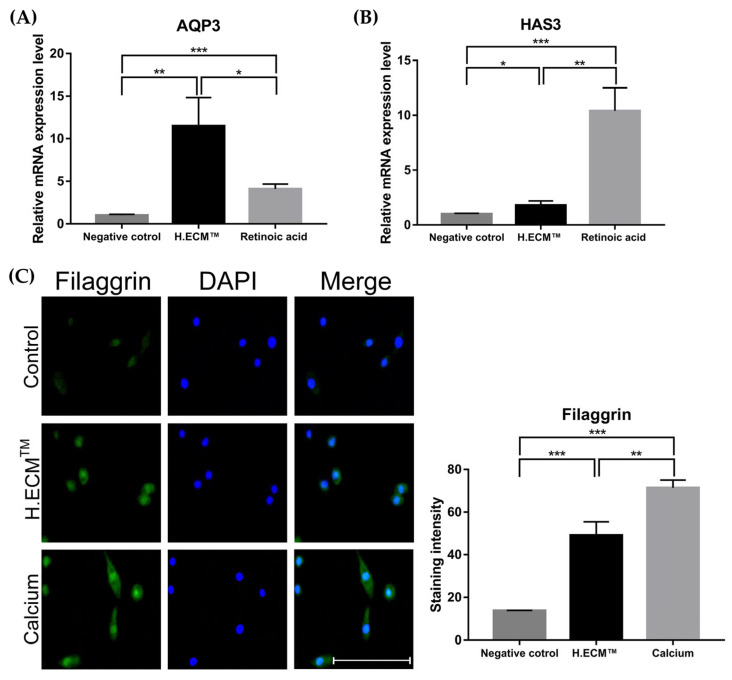
Expression levels of the *AQP3* and *HAS3* genes and the comparison of filaggrin expression via immunofluorescence staining using KCs. The expression of genes encoding skin hydration factors such as *AQP3* (**A**) and *HAS3* (**B**) were induced in the 0.05% H.ECM^TM^ liposome treatment group more than in the negative control group; 1 μM retinoic acid treatment was used as a positive control. Treatment with 0.05% H.ECM^TM^ liposome induced *filaggrin* expression more significantly than the negative control (**C**) 1.8 mM calcium treatment was used as a positive control. Scale bar indicated 100 μm. * *p* < 0.05, ** *p* < 0.01, *** *p* < 0.005.

Gene expression levels were evaluated using qRT-PCR. The expressions of the HAS3 and AQP3 genes involved in skin hydration were compared with those in response to treatment with 1 μM retinoic acid (positive control) using KCs. The expression levels of HAS3 and AQP3 in the 0.05% H.ECM^TM^ liposome treatment group increased significantly in comparison to those in the negative control group (* *p* < 0.05, ** *p* < 0.01, *** *p* < 0.005; Figure 3A,B). Notably, the expression level of AQP3 in the H.ECM^TM^ liposome-treated group was significantly higher than that in the positive control group (*** *p* < 0.005, Figure 3A). Additional immunofluorescence staining of filaggrin in KCs treated with 1.8 mM calcium (positive control) revealed that treatment with H.ECM^TM^ liposome significantly increased the expression level of filaggrin compared to that of the negative control group (** *p* < 0.01, *** *p* < 0.005; Figure 3C).

**Figure 4:**
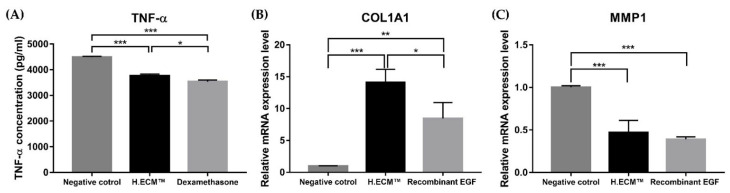
Variations in the expression levels of tumor necrosis factor (*TNF) –α* expression between the negative control, 1 μM Dexamethasone treatment and 0.05% H.ECM^TM^ liposome groups compared to the LPS-treated RAW 264.7 cells (**A**) Expression levels of *COL1A1* and *MMP1* genes. The expression levels of *COL1A1* (**B**) and *MMP1* (**C**), representing the expression of wrinkle-related factors, improved with 0.05% H.ECM^TM^ liposome treatment compared to the negative control group of HDF cells (**B**). The 10 ng/mL recombinant EFG treatment was used as a positive control. * *p* < 0.05, ** *p* < 0.01, *** *p* < 0.005.

Next, we performed ELISA to measure the TNF-α levels induced by the LPS treatment (2 μg/mL) of RAW 264.7 cells; dexamethasone (1 μM) was used as the positive control. When treated with H.ECM^TM^ liposome, the TNF-α levels were lower than in the negative control group (* *p* < 0.05, *** *p* < 0.005, Figure 4A). Collectively, these results suggest that H.ECM^TM^ liposome treatment enhances the skin barrier function and hydration and simultaneously exerts anti-inflammatory effects.

Lastly, the mRNA expression levels of the *COL1A1* and *MMP1* genes associated with skin aging and wrinkles were analyzed using HDF cells. Recombinant EGF was used as the positive control. In comparison with the negative control group, the expression level of *COL1A1* significantly increased in the H.ECM^TM^ liposome-treated group, while the expression of *MMP1* decreased, indicating that H.ECM^TM^ liposome treatment exerts anti-aging effects on skin and reduce wrinkles (** *p* < 0.01, *** *p* < 0.005, respectively; Figure 4B,C).

### 2.4. Ex Vivo Study on the Effect of H.ECM^TM^ Liposomes on Skin Barrier Function and Hydration

**Figure 5:**
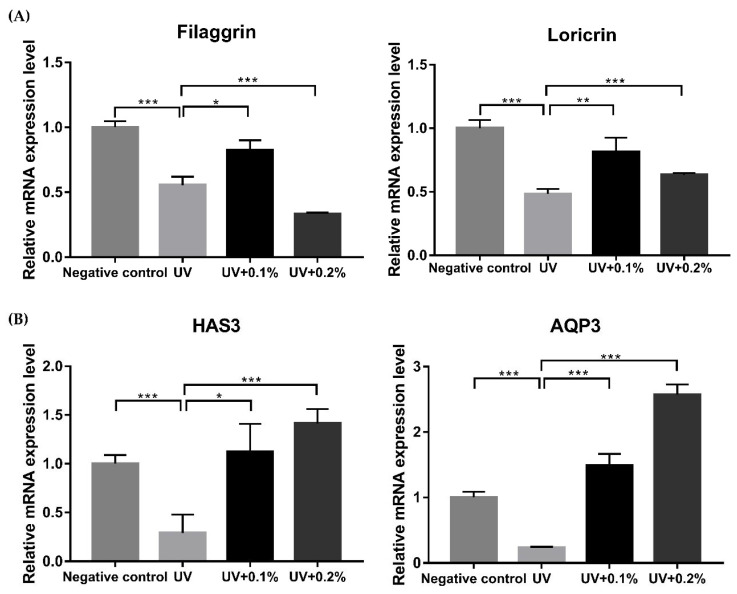
Expression levels of *filaggrin*, *loricrin*, *HAS3,* and *AQP3* genes in untreated tissue specimen (negative control), and only UVB irradiated (UV), 0.1% (UV + 0.1%) and 0.2% (UV + 0.2%) H.ECM^TM^ liposome treatment in UVB-irradiated tissue specimens. Expressions of genes encoding the factors related to the skin barrier function such as the *filaggrin* and *loricrin* were induced in the 0.1% and 0.2% H.ECM^TM^ liposome treatment groups more than the UV group (**A**). The expression levels of genes *COL1A1* and *MMP1* encoding skin hydration factors were higher in the 0.1% and 0.2% H.ECM^TM^ liposome treatments than the UV group (**B**). * *p* < 0.05, ** *p* < 0.01, *** *p* < 0.005.

The qRT-PCR and immunofluorescence staining were performed using an ex vivo model of human skin tissue to confirm the changes in the expression of the factors related to the skin barrier function and skin hydration upon application of the H.ECM^TM^ liposome. All experiments were conducted using three replicates of two different concentrations, 0.1%, and 0.2%. The qRT-PCR results indicated that UVB-irradiation significantly reduced the expression of not only filaggrin and loricrin but also the HAS3 and AQP3 genes associated with the skin barrier function (* *p* < 0.05, ** *p* < 0.01, *** *p* < 0.005; Figure 5). Treatment with 0.1% H.ECM^TM^ liposome enhanced the gene expression of filaggrin, loricrin, HAS3, and AQP3 (* *p* < 0.05, ** *p* < 0.01, *** *p* < 0.005; Figure 5A). Furthermore, treatment with 0.2% H.ECM^TM^ liposome after UVB-irradiation significantly increased the expression of loricrin, HAS3, and AQP3 (* *p* < 0.05, *** *p* < 0.005; Figure 5B).

**Figure 6:**
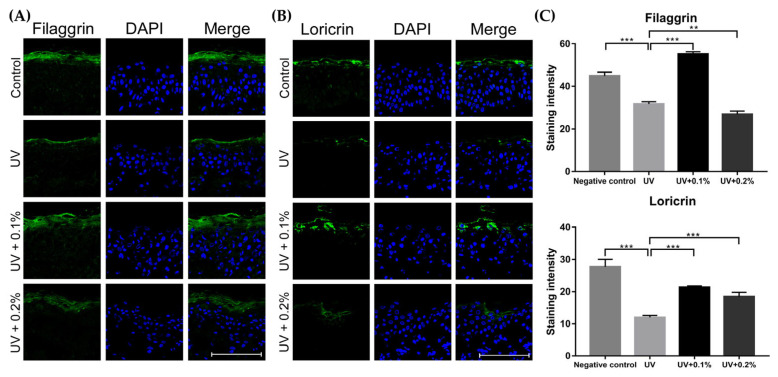
Comparison of filaggrin (**A**) and loricrin (**B**) expression using immunofluorescence staining. Treatments with 0.1% (UV + 0.1%) and 0.2% (UV + 0.2%) H.ECM^TM^ liposome increased the expression of filaggrin and loricrin that were reduced by UVB irradiation. Moreover, the expression after 0.1% H.ECM^TM^ liposome treatment was significantly higher than with 0.2% H.ECM^TM^ liposome (**C**). Scale bar indicated 100 μm. ** *p* < 0.01, *** *p* < 0.005.

In addition, the protein expression levels for filaggrin and loricrin proteins were measured via immunofluorescence staining (Figure 6A,B). Supplementary Figure S2 shows the results of the *t*-tests after qRT-PCR in the ex vivo study. After 300 mJ/cm^2^ UVB-irradiation, the filaggrin and loricrin expression was reduced, but increased significantly after treatment with 0.1% and 0.2% H.ECM^TM^ liposome (* *p* < 0.05, ** *p* < 0.01, *** *p* < 0.005; Figure 6C). Consistent with the qRT-PCR results, 0.1% H.ECM^TM^ liposome treatment enhanced filaggrin and loricrin expression more than the 0.2% H.ECM^TM^ liposome treatment. The results from the ex vivo study correlated well with the in vitro data, suggesting that H.ECM^TM^ liposome enhanced both the skin barrier function and hydration in damaged skin.

## 3. Discussion

Various topical emollients containing diverse, active ingredients have been used to treat dry, eczema-prone skin caused by atopic dermatitis and xerotic eczema [12]. However, limited information is available on the effect of soluble proteoglycans on restoring the skin barrier function via enhancement of hydration and reduction of skin inflammation. PGs and glycosaminoglycans (GAGs) are the major components of the extracellular matrix, along with collagen. HA is one of the known GAGs that form proteoglycan aggregates, which crosslink with other matrix proteins such as collagen, constituting supermolecular structures that increase skin firmness [13]. Previous studies on proteoglycans, HA, and collagen have focused on their anti-aging and anti-wrinkle effects [13,14,15,16,17,18,19,20].

Oral and topical formulations of collagen and HA have become increasingly available in the cosmeceutical market. Studies have shown that oral intake of collagen peptides attenuates UVB irradiation-induced skin dehydration by regulating HA synthesis [21]. Moreover, a clinical and ex vivo study showed that oral collagen peptide supplementation enhanced skin moisture and dermal collagen density [22]. A study in hairless mice also revealed that collagen hydrolysate intake mitigated the loss of epidermal barrier function and skin elasticity induced by ultraviolet radiation [23], confirming the role of collagen supplements in restoring the skin barrier function. Oral HA has also been shown to relieve wrinkles and dry skin in long-term clinical studies [24]. The effect of HA on collagen synthesis has also been explored in in vitro and in vivo studies [25].

In this study, we aimed to investigate the effect of a soluble proteoglycan combined with HA and hydrolyzed collagen on the restoration of the skin barrier function and hydration. We designed a four-week, single-arm prospective clinical trial of an emollient containing H.ECM^TM^ liposomes to observe its effect on mild atopic dermatitis and dry skin in participants. The participants showed statistically significant improvements in not only clinical symptoms (including itching), but also in objective biophysical parameters, including TEWL and the Corneometer measurement. The GIS evaluated at the end of the study revealed notable efficacy of the H.ECM^TM^ liposome-containing emollient on dry and eczema-prone skin.

Furthermore, our in vitro and ex vivo experiments revealed that the treatment of KCs with H.ECM^TM^ liposome promoted the mRNA expression of *HAS3* and *AQP3*, the factors influencing skin hydration. Immunofluorescence staining of KCs showed the increased expression of filaggrin upon treatment with the H.ECM^TM^ liposome-containing emollient, suggesting its role in enhancing the skin barrier function. The ex vivo experiments provided consistent results, revealing increased mRNA expression of *HAS3*, *AQP3*, *filaggrin*, and *loricrin* upon treatment with H.ECM^TM^ liposome in UVB-irradiated human skin. Immunofluorescence staining suggested that the expression of filaggrin and loricrin, the major constituent proteins of the epidermal skin barrier [26,27], also increased significantly after H.ECM^TM^ liposome treatment.

In addition, we investigated the difference in the expression of TNF-α upon H.ECM^TM^ liposome treatment using mouse macrophages. TNF-α is known to downregulate filaggrin and loricrin expression, thereby affecting the skin barrier function [28]. Treatment with H.ECM^TM^ liposome significantly decreased the expression of TNF-α, indicating its anti-inflammatory function and concomitantly improved the skin barrier. Lastly, in vitro experiments using HDFs showed significantly increased expression of *COL1A1* and *MMP-1*, implying anti-aging and anti-wrinkle formation effects of H.ECM^TM^ liposomes.

In conclusion, our clinical and laboratory experiments demonstrated the effects of H.ECM^TM^ liposomes on the restoration of the skin barrier function in atopic dermatitis and dry skin. Nonetheless, the study has several limitations. First, it was a single-arm study with a relatively small number of participants. Thus, randomized, placebo-controlled studies with larger populations are necessary to better advocate the benefits of the H.ECM^TM^ liposome-containing emollient. Secondly, although we have shown the changes in the expression of factors related to skin barrier function, hydration, and anti-aging via in vitro and ex vivo experiments, we did not explore the molecular pathways underlying changes in these factors. Further experimental studies need to investigate the pathophysiologic mechanism of H.ECM^TM^ liposomes in enhancing the skin barrier function and provide a complete understanding of the effect of topical proteoglycans combined with hydrolyzed collagen and HA on the skin.

## 4. Materials and Methods

### 4.1. Participants

The clinical study was conducted on 25 patients, aged 19 years or older, with mild atopic dermatitis and dry skin, under informed consent. The study was conducted after receiving approval from the Clinical Trial Review Committee of Severance Hospital, Yonsei University College of Medicine (IRB number 1-2020-0063), in compliance with the ethical principles of the Declaration of Helsinki. No topical or systemic anti-inflammatory treatments were administered 2 weeks prior to the inclusion of the patients in the study. The inclusion criteria included patients previously diagnosed with mild atopic dermatitis (Eczema Area and Severity Index < 15) or dry skin with a Xerosis Assessment Scale (XAS) [29] greater than 2 (0: absence of xerosis; 1: a few minute flakes, 2: many undifferentiated skin flakes, 3: some polygonal scales; 4: a moderate number of polygonal scales; 5: a large number of polygonal scales; 6: fissuring between scales; 7: moderate deep fissuring between scales; 8: deep fissuring). Exclusion criteria included a history of uncontrolled medical illness, pregnancy, and any form of dermatologic treatment (e.g., laser, filler, or tattoos) in the 3 months preceding the study. Patients with atopic dermatitis who underwent systemic anti-inflammatory or immunosuppressive treatments within 1 month and those that received topical treatments or oral antihistamines within 2 weeks of the study were also excluded.

### 4.2. Application of an H.ECM^TM^ Liposome-Containing Emollient Containing Proteoglycan Combined with Hyaluronic Acid and Collagen

The topical emollient investigated in this study contained 0.01% H.ECM^TM^ liposomes, a novel product composed of a soluble proteoglycan (900–1200 kDa) derived from salmon nasal cartilage, emulsified with HA on its non-denatured N-terminus, and hydrolyzed collagen. This composition was prepared in the form of nano-liposomes, which can easily penetrate the skin with a diameter of 50–150 nm. The 25 participants included in the study consented to apply the moisturizer to the skin lesions at least twice daily for 4 weeks to evaluate the safety and efficacy of this emollient. To assess the safety, all participants were asked to report any adverse events experienced while using the topical emollient.

### 4.3. Assessments for Clinical Efficacy

All patients were followed up at baseline, week 2, and week 4 after the initiation of treatment. Standardized digital photographs were taken (Canon EOS 850D, Tokyo, Japan) at each visit, and three independent investigators scored the outcomes on a 5-point scale, GIS (grade 1, more than 75% = near-total improvement; grade 2, 51–75% = marked improvement; grade 3, 26–50% = moderate improvement; grade 4, 0–25% = minimal improvement; grade 5 = no improvement) and the subjects’ GIS were obtained based on the clinical photographs at week 4. The clinical efficacy of the study product was also investigated by biophysical parameters of TEWL, skin hydration, and skin erythema at each visit. The skin barrier function and hydration were evaluated using a Corneometer^®^ and Tewemeter^®^ (Courage Khazaka Electronic GmbH, Köln, Germany), while skin erythema was measured using a Mexameter^®^ MX 18 (Courage Khazaka Electronic GmbH, Köln, Germany). The measurements were conducted after subjects were acclimatized to the environment for 30 min at each visit under controlled environmental conditions (room temperature: 18–21 °C relative humidity: 40–60%, without direct light). Finally, the symptomatic improvement in the degree of itching was evaluated at each visit by the participants via VRS (grade 0 = no itching, grade 1 = mild itching, grade 2 = moderate itching, grade 3 = severe itching, 4 = very severe itching).

### 4.4. Cell Culture

A mouse macrophage (RAW 264.7), a human epidermal keratinocyte (KC), and human dermal fibroblast (HDF) cell lines were procured from ATCC^®^ (Manassas, VA, USA) and Thermo Fisher Scientific (Waltham, MA, USA). RAW 264.7 cells were cultured in Dulbecco’s modified Eagle’s Medium (DMEM; Lonza, Walkersville, MD, USA) containing 10% (*v/v*) fetal bovine serum (FBS; Gibco, Grand island, NY, USA) and 1% Penicillin-Streptavidin (PS; Gibco) and KCs were cultured in KBM^TM^ Gold Basal Medium (Lonza) containing KGM^TM^ Gold SingleQuots^TM^ supplements (Lonza). HDF cells were cultured in Roswell Park Memorial Institute Medium (RPMI) 1640 (Lonza) supplemented with 10% FBS and 1% PS. All cells were incubated at 37 ℃ in a humidified atmosphere containing 5% CO_2_.

### 4.5. Cell Cytotoxicity Measurement

Cell cytotoxicity was measured using the MTT assay. Briefly, each cell line (1 × 10^4^ cells/well) was cultured in 48-well cell culture plates and treated with H.ECM™ liposome (0.01–1%) when cells reached over 80% confluence. Additionally, HDF cells were treated with LPS (0.5–50 μg/mL). After treatment, 200 μL MTT solution (diluted in each medium; 0.5 mg/mL) was added to the wells, and the cells were incubated at 37 °C for 4 h. Next, 200 μL dimethyl sulfoxide (DMSO) was added to each well, and 100 μL sample aliquot was transferred to a 96-well plate. The absorbance at 585 nm was measured using an ELISA microplate reader (VersaMax; Molecular Devices, California, CA, USA).

### 4.6. Enzyme-Linked Immunosorbent Assay

RAW 264.7 cells (2 × 10^4^ cells/well) were seeded in 12-well plates and treated with LPS (2 μg/mL; Sigma-Aldrich, Saint Louis, MO, USA) for inducing TNF-α expression when cells reached more than 80% confluence. The cells were treated 0.1% H.ECM™ liposome or 1 μM Dexamethasone (positive control; Sigma-Aldrich) and incubated for 24 h. Then, the media used at culture was centrifuged at 2000× *g* for 10 min, and the supernatant was used for ELISA. The Mouse TNF-α ELISA kit (Abcam, Cambridge, MA, USA) was used for TNF-α measurement. ELISA was performed according to the manufacturer’s instructions. The absorbance at 450 nm was measured using an ELISA microplate reader (VersaMax; Molecular Devices, California, CA, USA).

### 4.7. Tissue Culture

The clinical trial was approved by the Institutional Review Board of Severance Hospital, Yonsei University (IRB No. 4-2021-0262). For tissue culture, subcutaneous fat was removed from human skin tissue, and the tissue was cut into 1 cm × 1 cm pieces. The tissue specimens were placed into 6-well plates containing semi-solid DMEM and UVB-irradiated at a dose of 300 mJ/cm^2^ using a UVB lamp (BLX26, BIO-LINK^®^-Crosslinker, FRA). The surface of the irradiated tissue specimen was treated with 20 μL H-liposome at 0.1% and 0.2% concentrations and incubated at 37 ℃ in a humidified atmosphere containing 5% CO_2_. The irradiation was conducted three times every 24 h. Each tissue specimen used in the present experiment was obtained after 24 h of additional incubation.

### 4.8. Quantitative Reverse Transcription PCR

KCs (2 × 10^4^ cells/well) and HDF cells (2 × 10^4^ cells/well) were seeded into 12-well plates and treated with 0.05% H.ECM™ liposome or positive control (1 μM retinoic acid (Sigma-Aldrich) or 10 ng/mL recombinant epidermal growth factor (EGF; R&D systems, Minneapolis, MN, USA) when the cells reached around 80% confluence. Then, the UVB-irradiated human skin specimens were homogenized using TissueLyser Ⅱ (Qiagen, Hilden, Germany). Total RNA was extracted using the RNAiso Plus (Invitrogen, Waltham, MA, USA) according to the manufacturer’s instructions. Total RNA was quantified using a NanoDrop 2000 spectrophotometer (ThermoFisher) and reverse transcribed using an RNA to cDNA EcoDry Premix Kit (Takara Sake, Berkley, CA, USA). The synthesized cDNA, Taqman Gene Expression Master Mix (Applied Biosystems), and Taqman primer of each target (*HAS3*; Hs00193436_m1, *AQP3*; Hs00185020_m1, *COL1A1*: Hs00164004_m1, *MMP1*: Hs00899658_m1; *Filaggrin*; Hs00856927_g1, *Loricrin*; Hs01894962_s1, Applied Biosystems) were used for the qRT-PCR experiments. The expression level of each gene was normalized to that of the housekeeping gene *GAPDH* (Hs02786624_g1, Applied Biosystems) and calculated using the 2^−ΔΔCt^ method.

### 4.9. Immunofluorescence Staining

In 8-well chamber slides, KCs (1 × 10^4^ cells/well) were seeded and treated with the positive control agent (1.8 mM calcium) or 0.05% H.ECM™ liposome when they reached over 80% confluence and incubated for 24 h. For immunofluorescence, the 8-well chamber slide containing treated KC cells and human skin cryosections (6 μm-thick) were fixed at room temperature (20–25 °C) with 4% (*w/v*) paraformaldehyde (Cell Signaling Technology, Danvers, MA, USA) for 15 min. Post-fixation, the chamber or cryosections were washed with phosphate-buffered saline (PBS)-T (PBS containing Triton X-100; DAEJUNG, Busan, KR) three times, followed by incubation with anti-filaggrin (ab81468; Abcam) or nti-loricrin (ab85679; Abcam) antibodies overnight at 4 °C. After washing three times with PBS-T, samples were incubated with the FITC-labeled secondary fluorescent antibody (Goat pAb to Rb IgG-FITC, ab6717; Abcam) for 2 h at room temperature to bind primary antibodies. Finally, the cells and tissue specimen were fixed using the fixation solution (VECTASHIELD^®^ mounting medium with 4′,6-diamidino-2-phenylindole (DAPI; Vector Laboratories Inc., Burlingame, CA, USA). All fluorescence images were acquired using a laser-scanning microscope (LSM 700, Carl Zeiss, Jena, Germany), and fluorescence intensity was measured using ImageJ software (National Institutes of Health, Bethesda, MD, USA).

### 4.10. Statistical Analysis

Data are presented as numbers (percentages) or means ± standard deviations. Data sets were assessed for normality using the Kolmogorov–Smirnov test. Repeated-measures analysis of variance and subsequent *post hoc* analysis by the Student’s *t*-test were performed with Bonferroni’s correction to account for multiple comparisons. A *p*-value < 0.05 was considered significant (* *p* < 0.05, ** *p* < 0.01, *** *p* < 0.005). All statistical analyses were performed using SPSS version 25.0 (IBM Corp., Armonk, NY, USA). All laboratory experiments were conducted at least three times (*n* ≥ 3), and the collected data was used for statistical analyses.

## Supplementary Materials

The following are available online at https://www.mdpi.com/article/10.3390/ijms221910189/s1.
