# Supplementary material for: Proteoglycan Combined with Hyaluronic Acid and Hydrolyzed Collagen Restores the Skin Barrier in Mild Atopic Dermatitis and Dry, Eczema-Prone Skin: A Pilot Study
Source: Int J Mol Sci. 2021 Sep 22;22(19):10189. doi: 10.3390/ijms221910189 (PMC8508667; doi:10.3390/ijms221910189)
Supplement: Supplementary file 1 [file ijms-22-10189-s001.zip › hugel_proteoglycan_suppl.pdf]

International Journal of *Molecular Sciences*

MDPI

Supplementary Materials

Proteoglycan combined with hyaluronic acid and hydrolyzed collagen restores skin barrier in mild atopic dermatitis and dry, eczema-prone skin

Young In Lee [1,2,†], Sang Gyu Lee [1,†], Jemin Kim [1,2], Sooyeon Choi [1], Inhee Jung [3] and Ju Hee Lee [1,2,*]

[1] Department of Dermatology & Cutaneous Biology Research Institute, Yonsei University College of Medicine, Seoul, Korea; ylee1124@yuhs.ac (Y.I.L.); dltkdrb5658@yuhs.ac (S.G.L.); JEMIN89ZZ@yuhs.ac (J.K.); CHOISY429@yuhs.ac (S.C.)
[2] Scar Laser and Plastic Surgery Center, Yonsei Cancer Hospital, Seoul, Korea
[3] Global Medical Research Center, Seoul, Korea; injung@gmrc.co.kr

* Correspondence: juhee@yuhs.ac; Tel.: +82-2-2228-2080

1. Supplementary Table

Table S1. Patient demographics.

Screening No.	Sex	Age	Diagnosis	Measurment Lesions	XAS (xerosis assessment scale)[1]
S01	F	22	Atopic dermatitis, mild	Lt.antecubital fossa	N/A
S02	F	56	Xerosis cutis	Lt.upper arm	2
S03	F	40	Xerosis cutis	Rt.lower leg	2
S04	F	51	Xerosis cutis	Rt.lower arm	5
S05	F	43	Xerosis cutis	Rt.lower leg	2
S06	F	40	Xerosis cutis	Rt. Hand	3
S07	F	52	Atopic dermatitis, mild	posterior neck	N/A
S08	F	46	Xerosis cutis	Rt. Hand	2
S09	F	33	Xerosis cutis	Rt. Hand	2
S10	F	51	Xerosis cutis	Rt.lower leg	4
S11	F	22	Xerosis cutis	Lt. Hand	2
S12	M	23	Atopic dermatitis, mild	Lt.antecubital fossa	N/A
S13	F	41	Atopic dermatitis, mild	Lt.upper arm	N/A
S14	F	45	Xerosis cutis	Rt.lower leg	4
S15	F	56	Xerosis cutis	Rt. Hand	3
S16	F	47	Xerosis cutis	Rt.lower leg	4
S17	F	39	Xerosis cutis	Rt.lower leg	5
S18	F	48	Xerosis cutis	Rt.lower arm	2
S19	M	47	Atopic dermatitis, mild	Lt.lower leg	N/A
S20	F	48	Xerosis cutis	Lt.lower leg	2
S21	F	20	Atopic dermatitis, mild	Rt.antecubital fossa	N/A

S22	M	56	Xerosis cutis	Lt. foot	2
S23	M	38	Xerosis cutis	posterior neck	2
S24	F	28	Xerosis cutis	Rt.lower leg	5
S25	F	26	Xerosis cutis	Rt.lower arm	2

[1.] XAS 0: absence of xerosis; 1: a few minute flakes, 2: many undifferentiated skin flakes, 3: some polygonal scales; 4: a moderate number of polygonal scales; 5: a large number of polygonal scale; 6: fissuring between scales; 7: moderate deep fissuring between scales; 8: deep fissuring.

2. Supplementary Figure

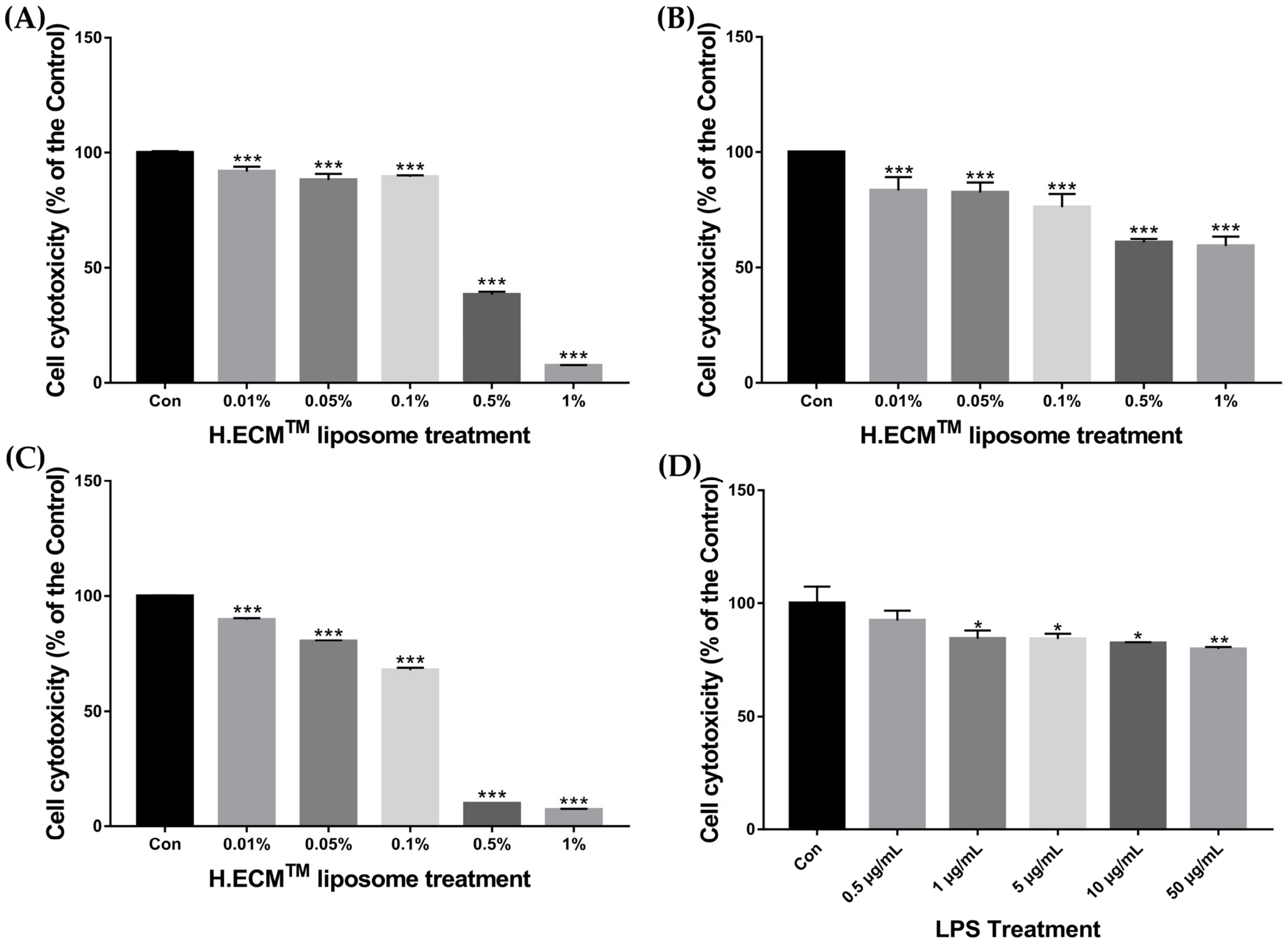

Figure S1. Cell cytotoxicity of H.ECM™ liposome and LPS depending on concentration. Concentration-dependent cell cytotoxicity of H.ECM™ liposome was conformed on RAW264.7 cells (A), KC cells (B) and HDF cells (C). Concentration-dependent cell cytotoxicity of LPS was conformed on HDF cells (D). (*$p < 0.05$, ** $p < 0.01$, *** $p < 0.005$).

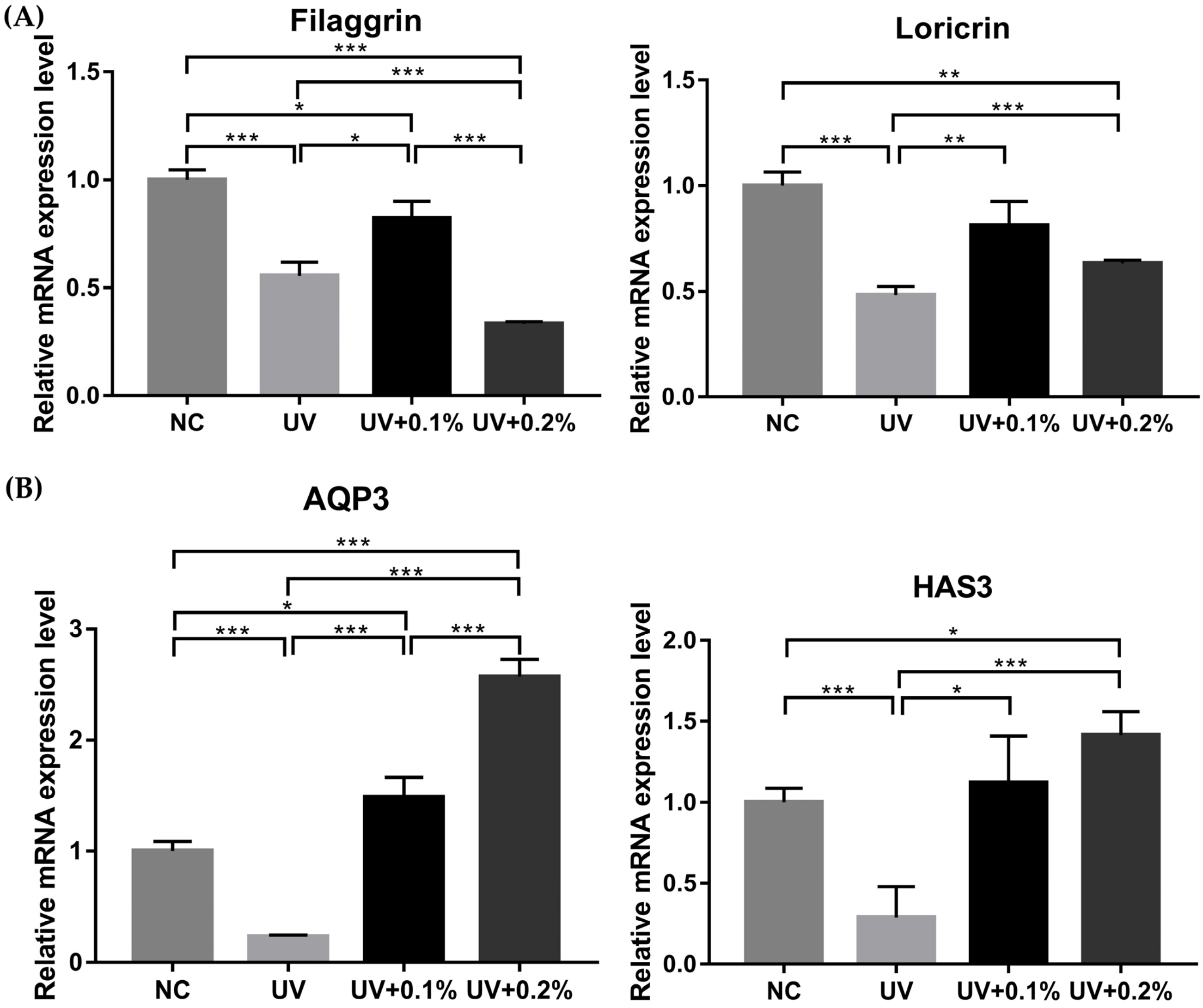

Figure S2. Expression levels of filaggrin, loricrin (A), HAS3 and AQP3 (B) gene with un-treatment at tissue specimen (negative control), only UVB irradiation (UV), 0.1% (UV+0.1%) and 0.2% (UV+0.2%) H.ECM™ liposome treatment at UVB-irradiated tissue specimen. (*$p < 0.05$, **$p < 0.01$, ***$p < 0.005$).
