# Supplementary figures and images for: Proteoglycan Combined with Hyaluronic Acid and Hydrolyzed Collagen Restores the Skin Barrier in Mild Atopic Dermatitis and Dry, Eczema-Prone Skin: A Pilot Study
Source: Int J Mol Sci. 2021 Sep 22;22(19):10189. doi: 10.3390/ijms221910189 (PMC8508667; doi:10.3390/ijms221910189)

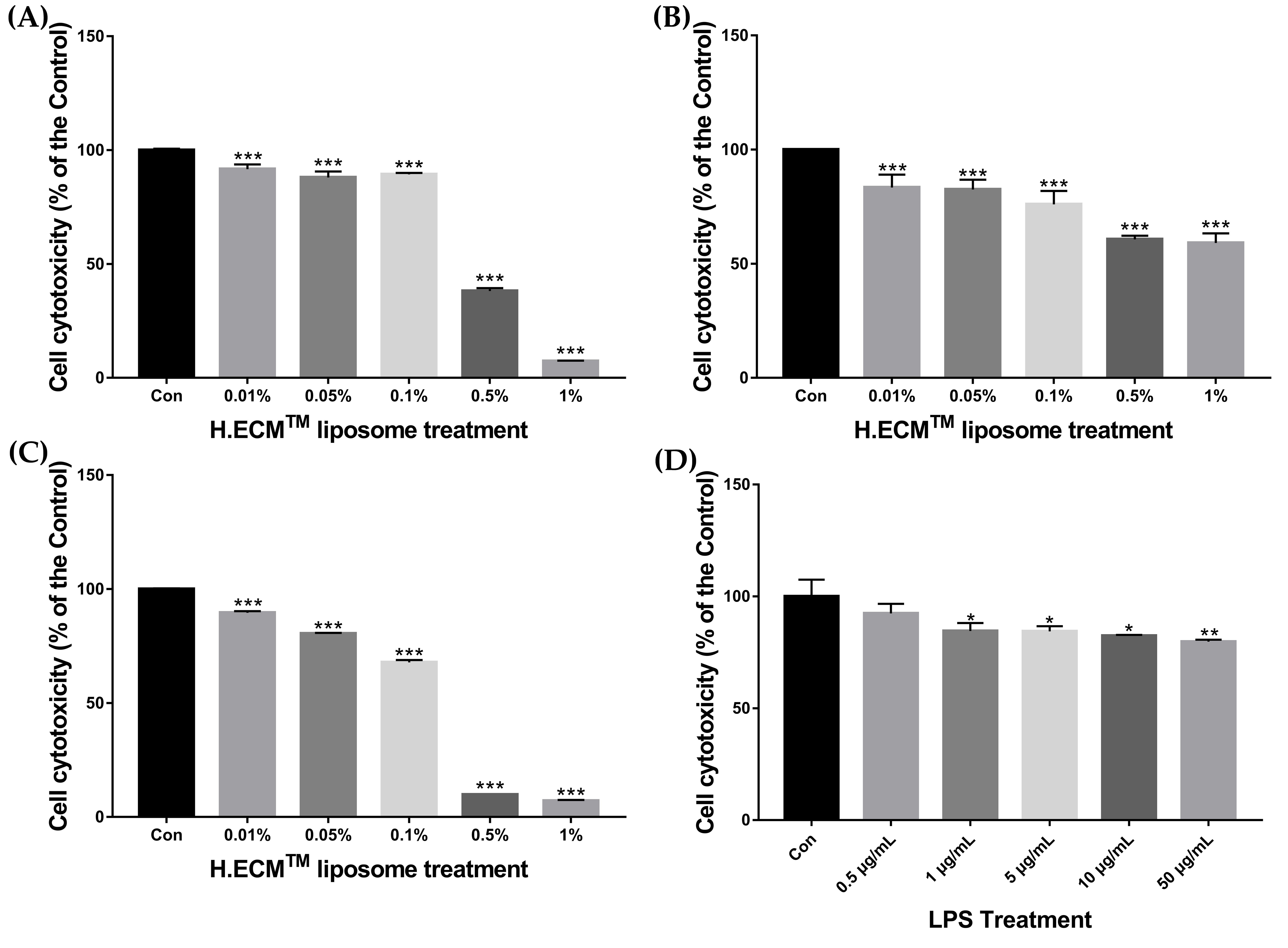

Supplement: Supplementary file 1 [file ijms-22-10189-s001.zip › Supplementary figure 1.png]

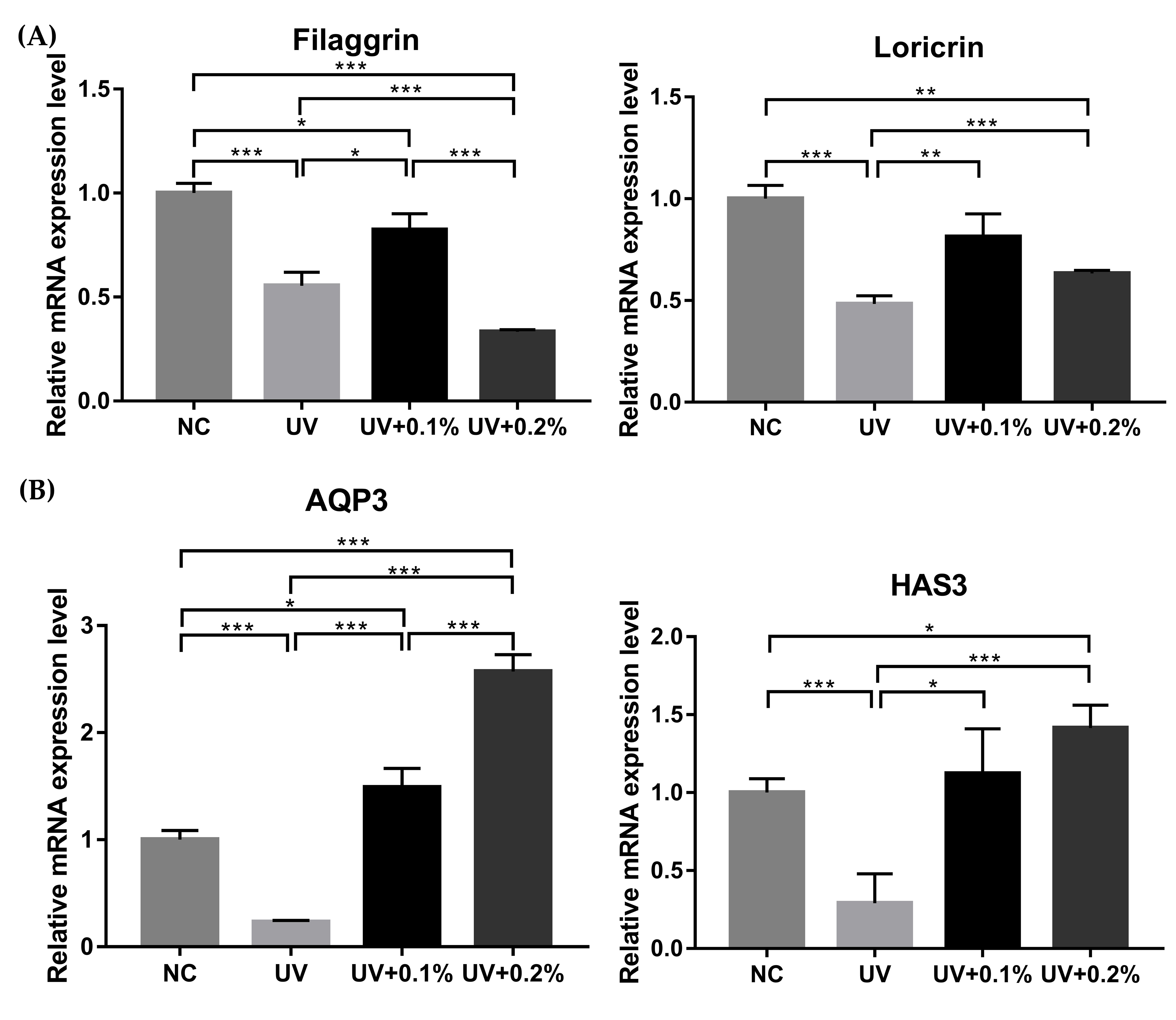

Supplement: Supplementary file 1 [file ijms-22-10189-s001.zip › Supplementary figure 2.png]
